# Pharmacological Therapies and Their Clinical Targets in Irritable Bowel Syndrome With Diarrhea

**DOI:** 10.3389/fphar.2020.629026

**Published:** 2021-02-18

**Authors:** Esther Colomier, Joost Algera, Chloé Melchior

**Affiliations:** ^1^ Department of Molecular and Clinical Medicine, Institute of Medicine, Sahlgrenska Academy, University of Gothenburg, Gothenburg, Sweden; ^2^ Translational Research Center for Gastrointestinal Disorders (TARGID), Department of Chronic Diseases, Metabolism and Aging (CHROMETA), KU Leuven, Leuven, Belgium; ^3^ Gastroenterology Department and INSERM CIC-CRB 1404, Rouen University Hospital, Rouen, France; ^4^ INSERM UMR 1073, Institute for Research and Innovation in Biomedicine, Normandy University, Rouen, France

**Keywords:** diarrhea—therapy, pharmacology, irritable bowel syndrome with diarrhea, clinical management algorithms, abdominal pain, loose stools, bloating, irritable bowel syndrome (IBS)

## Abstract

Irritable bowel syndrome (IBS) is one of the most common disorders of the gut-brain axis, which affects approximately 4% of the global population. The Rome IV criteria define IBS as chronic or recurrent abdominal pain associated with altered bowel habits. Patients can be categorized in four subtypes: IBS with predominant constipation (IBS-C), predominant diarrhea (IBS-D), mixed bowel habits (IBS-M), and unclassified (IBS-U). IBS is associated with a lower quality of life, reduced work productivity, and high healthcare costs. When comparing subtypes, patients with IBS-D report lower disease related quality of life. Due to the scope of this review, we have solely focused on patients with IBS-D. Choosing the right pharmacological treatment in these patients remains challenging due to the heterogeneous patient population, patients’ expectation of the treatment outcome, unavailability of efficacious drugs, and the multifactorial and incompletely understood underlying pathophysiology. Currently, pharmacological treatment options target individual symptoms, such as abdominal pain, altered bowel habits, and bloating. In this review, we aimed to summarize the current and recent pharmacological treatment options in IBS-D, targeting the predominant gastrointestinal symptoms. Additionally, we proposed a pharmacological treatment algorithm which healthcare professionals could use when treating individual patients with IBS-D.

## Introduction

With a worldwide prevalence of approximately 4%, irritable bowel syndrome (IBS) is one of the most common functional gastrointestinal disorders (FGIDs) (Sperber et al., 2021). These FGIDs have recently been renamed to disorders of the gut-brain axis (DGBIs). IBS is ranked as one of the most common reasons for a consultation in primary care and a referral to secondary or tertiary care in gastroenterology (Drossman, 2016). The prevalence of 4% is obtained using the current Rome IV diagnostic criteria. They define IBS as recurrent abdominal pain associated to at least two of the following items; defecation, a change in stool frequency or a change in stool form. On average, this recurrent abdominal pain should have occurred at least one day per week in the last three months and symptoms must have been present for at least six months (Lacy et al., 2016). Apart from the observed abdominal pain and abnormal bowel habits, frequently reported symptoms include abdominal distention, bloating, and flatulence (Lacy et al., 2016). Based on the predominant stool type, assessed with the Bristol stool form scale (BSFS) (Lewis and Heaton, 1997), IBS patients can be categorized into four subgroups: IBS with predominant constipation (IBS-C), IBS with predominant diarrhea (IBS-D), IBS with mixed bowel habits (IBS-M), and unclassified IBS (IBS-U) (Lacy et al., 2016). However, over time, symptoms can differ and patients can move from one entity to another.

Currently, there are no objective biomarkers available for IBS to guide treatment. Routine clinical investigations do not indicate organic abnormalities that can explain the symptoms. The well-established pathophysiological factors include visceral hypersensitivity (Simrén et al., 2018), altered gastrointestinal (GI) motility (Manabe et al., 2010; Törnblom et al., 2012), disturbances in gut-brain interaction, and psychological distress. More recently, research is demonstrating and further investigating alterations in the microbiome, intestinal immune activation, increased intestinal permeability, and food hypersensitivity with a focus on gut luminal factors (Simrén et al., 2001; Öhman et al., 2015; Barbara et al., 2016; Bednarska et al., 2017; Tap et al., 2017). However, not all these factors are seen in every patient. Therefore, one should consider heterogeneity when assessing the clinical characteristics in IBS.

Due to the incompletely elucidated pathophysiology and the heterogeneity among the symptom profiles of IBS patients, the pharmacological treatment options target the most common individual IBS symptoms; i.e., abdominal pain, altered bowel habits, and bloating. Therefore, treating patients is often described as a process of trial-and-error. No treatment option fits all patients, making the management of these patients very versatile and complex. Due to the more recent focus on the gut luminal factors, such as food and the microbiome, dietary interventions also became more important (Algera et al., 2019).

In this review, we will focus on the pharmacological treatment options for patients with IBS-D, targeting GI motility, visceral hypersensitivity, and altered gut luminal factors. Research shows that patients with IBS-D have a lower disease-related quality of life compared to patients with IBS-C, which impacts work productivity and daily life activities (Singh et al., 2015; Buono et al., 2017). Fecal urgency is considered the most troublesome symptom. In general, most IBS-D patients regularly use multiple treatments that are unsatisfactory, and report substantial psychological burden (Törnblom et al., 2018). Higher severity scores are also associated with increased medication use and a worse attitude toward the condition itself (Emmanuel et al., 2020). This should all be considered in the management of these patients. Healthcare professionals understand the high symptom burden IBS-D patients experience, but still find the condition difficult to treat (Törnblom et al., 2018).

Therefore, we have aimed to summarize the first- and second-line pharmacological treatment options in patients with IBS-D. Due to their good accessibility, popularity, and applicability in patients with severe comorbidities or contraindications to pharmacological treatment, we also aimed to summarize ‘probiotics and plant-derived product’. In addition, we proposed a pharmacological treatment algorithm for healthcare professionals, which could provide guidance in working toward an individualized pharmacological management of patients with IBS-D.

## Methods

This review is based on literature searches performed in the PubMed database in October 2020 using the search terms “irritable bowel syndrome”, “functional bowel disorder”, “diarrhea”, “therapy”, “treatment”, “randomized clinical trial”, “probiotics”, “antibiotics”, “antispasmodics”, “peppermint oil”, “herbal”, “plant-derived”, “antidepressants”, “loperamide”, “cholestyramine”, “alosetron”, “ramosetron”, “ondansetron”, “rifaximin”, “eluxadoline”, “xyloglucan”, “crofelemer”. In the identified articles, reference lists were used to add additional papers. Both clinical research and review articles in English were considered, without restrictions regarding publication year. Papers about IBS in children, case reports, case–controlled studies were excluded.

### First-Line Pharmacological Treatments

#### Antispasmodics Targeting Abdominal Pain

For patients who experience abdominal pain, antispasmodics have been the first-line treatment option in primary care for decades. Depending on the agent, the mechanism of action is related to their anticholinergic and calcium channel blocking properties leading to smooth muscle relaxation in the gut (Annaházi et al., 2014). A subgroup of IBS patients, and especially IBS-D patients, have an exaggerated gastro-colonic reflex that is partially mediated by a cholinergic pathway (Chey et al., 2001). Therefore, antispasmodics might be best suited for patients with abdominal cramps and altered bowel habits. Frequently used examples are: alverine citrate (+simethicone), mebeverine, otionium bromide, pinaverium bromide, and phloroglucinol. Table 1 gives an overview of the most-described randomized controlled trials (RCTs) assessing the effect of different antispasmodics in approximately 2900 IBS patients. In general, placebo-controlled trials show only low-quality efficacy evidence and most often only short-term symptom relief (Tack et al., 2006b; Ford et al., 2008; Annaházi et al., 2014). Antispasmodics are relatively safe, but one should be aware of the potential anticholinergic side effects and contraindications. The anticholinergic side effects include constipation, dry mouth, visual disturbances, and urinary retention. Most common contraindications include glaucoma, GI obstruction, autonomic neuropathy, obstructive uropathy, and patients with an allergy for barbiturates, Belladonna Alkaloids or antiepileptics with arene oxide metabolites. Due to the anticholinergic side effects, their usage in elderly, patients with benign prostate hyperplasia, glaucoma, urinary bladder neck obstruction, myasthenia gravis, and Alzheimer’s disease is most often problematic.

**Good candidates for antispasmodic treatment (first-line)** Mild or moderate IBS patients presenting cramps and/or intermittent abdominal pain as the predominant symptom.

**TABLE 1:** Randomized controlled trials evaluating the efficacy of antispasmodics in IBS patients with diarrhea.

**Study**	**Population** (***n***)*,* **Rome criteria**	**Period (weeks)**	**Dose**	**Main outcome, significant difference compared to placebo**	**Adverse events**
**Cimetropium bromide**					
Centonze et al. (1988), Italy	IBS (*n* = 48), no Rome criteria	24	50 mg t.i.d	Decrease in pain scores (RR: 87% vs. 16%)	Dry mouth and sleepiness
Dobrilla et al. (1990), Italy	IBS (*n* = 70, 35 cimetropium bromide), no Rome criteria	12	50 mg t.i.d	Decreased severity pain scores (RR: 85% vs. 52%)	Dry mouth
**Drotaverine hydrochloride**					
Rai et al. (2014), India	IBS (*n* = 170, 85 drotaverine HCl), Rome II	4	80 mg t.i.d	Decreased pain frequency and severity scores (RR: 78% vs. 31%)	Headache, heartburn, flatulence
**Phloroglucinol (+ Trimethylphloroglucinol)**					
Chassany et al. (2007), France	IBS with acute abdominal pain (*n* = 300, 151 P + TMP), Rome II	1	62.2 mg P + 80 mg TMP t.i.d	Decrease in pain intensity (RR: 60% vs. 32%)	Constipation, flatulence, and abdominal pain
Shin et al. (2020), Korea	IBS-D (*n* = 72, 36 P), Rome III	3	160 mg t.i.d	Global symptom improvement (RR: 62% vs. 31%)	Nausea
**Otilonium bromide**					
Glende et al. (2002), Italy	IBS (*n* = 317, 160 OB), Rome I	15	40 mg t.i.d	More frequent improvement of GI symptoms (RR: 37% vs. 23%)	None related to the study medication
Clavé et al. (2011), Spain	IBS (*n* = 356, 179 OB), Rome II	25	40 mg t.i.d	Reduction in the number of abdominal pain episodes. (−0.90 ± 0.88 vs. −0.65 ± 0.91)	Dry mouth and nausea
Battaglia et al. (1998), Italy	IBS (*n* = 325, 160 OB), no Rome criteria	15	40 mg t.i.d	Reduced frequency of abdominal pain episodes (RR: 55% vs. 40%)	Not reported
**Mebeverine**					
Kruis et al. (1986), Germany	IBS (*n* = 120, 40 mebeverine), no Rome criteria, mixed	16	400 mg o.d	No significant symptomatic improvement	Not reported
Everitt et al. (2010), United Kingdom	IBS (*n* = 135, 44 mebeverine), Rome III	6	135 mg t.i.d. vs. b.i.d	No significant differences in IBS-SSS score	None related to the study medication
**Pinaverium bromide**					
Zheng et al. (2015), China	IBS-D (*n* = 427, 218 pinaverium bromide), Rome III	4	50 mg t.i.d	Improved abdominal pain (RR: 62% vs 30%)	Nausea, dizziness, increased BP, abdominal discomfort
**Alverine citrate**					
Ducrotte et al. (2014), France	IBS (*n* = 436, 222 alverine citrate/simethicone), Rome III	24	60 mg + 300 mg t.i.d	Decreased total score IBS-Severity Scoring System (170 vs. 111)	Not reported
Wittmann et al. (2010), Hungary	IBS (*n* = 409, alverine citrate-simethicone), Rome III	4	60 mg + 300 mg t.i.d	50% decrease in abdominal pain/discomfort VAS scores (RR: 47% vs. 34%)	Not reported

b.i.d., twice daily; BP, blood pressure; IBS, irritable bowel syndrome; IBS-D, IBS with predominant diarrhea; o. d., once daily; P, phloroglucinol; RR, response rate; t. i.d., thrice daily; TMP, trimethylphloroglucinol; VAS, visual analogue scale.

#### Peppermint Oil Targeting Abdominal Pain

**FIGURE 1:**
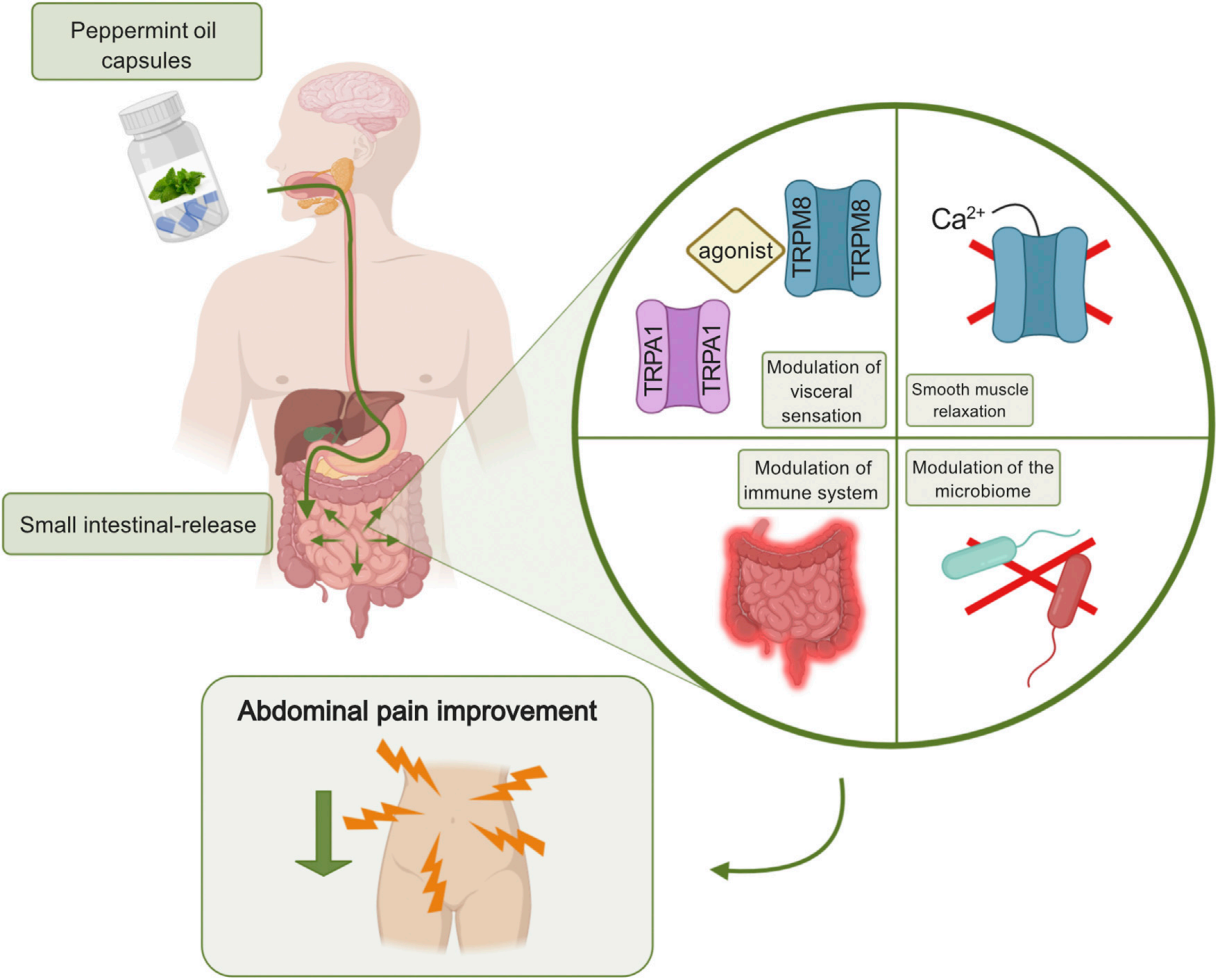
Peppermint oil targeting abdominal pain in IBS. Enteric-coated peppermint oil capsules are released in the small intestine causing a modulation of visceral sensation, smooth muscle relaxation, a modulation of the immune system, and a modulation of the microbiome. The agent is anti-viral and anti-fungal. All proposed mechanisms of action can lead to a decrease in abdominal pain perception. Created with BioRender.com.

One of the safer, more “natural”, agents with antispasmodic properties is peppermint oil with the active ingredient, L-menthol. Apart from its antispasmodic effect, research remains rather unclear about the additional beneficial properties of L-menthol. This over-the-counter relaxant induces a blockade of L-type calcium channels without activating transient receptor potential cation channel subfamily M member 8 (TRPM8) channel or nitrous oxide (Hills and Aaronson, 1991). Its analgesic characteristics might also be related to the effect on the transient receptor potential cation channel, subfamily A, member 1 (TRPA1), in the cells of Cajal, inducing concentration-dependent membrane potential depolarization (Chumpitazi et al., 2018) (Figure 1). Furthermore, it has been shown that peppermint oil is antimicrobial, antifungal, and anti-viral, most often targeting obligate and facultative anaerobes and enteric pathogens. The agents also appear to be anti-inflammatory by suppressing the production of inflammatory mediators originating from monocytes (Chumpitazi et al., 2018). Table 2 describes RCTs that investigated the efficacy of peppermint oil in approximately 500 IBS patients. A number of RCTs suggested that enteric peppermint oil has a positive effect on IBS patients by relieving abdominal pain and discomfort, and global IBS symptoms after 4 weeks of treatment (Cappello et al., 2007; Merat et al., 2010; Alammar et al., 2019; Black et al., 2020b). Earlier studies do not provide high-quality data regarding the long-term efficacy. Only the more recent formulation of peppermint oil with small-intestinal-release demonstrated to significantly reduce IBS severity, abdominal pain and discomfort, bloating, and urgency (Cash et al., 2016; Weerts et al., 2020). The one with ileocolonic-release failed to demonstrate any efficacy compared to placebo (Weerts et al., 2020). Heartburn, urine and/or feces that smell like menthol, and nausea are common side effects of peppermint oil. The usage of peppermint oil is contraindicated in patients with severe liver, gallbladder or bile ducts disease, and in patients who are hypersensitive or allergic to menthol.

**Good candidates for the treatment with peppermint oil (first-line)** Moderate IBS-D patients with permanent (or intermittent) abdominal pain and discomfort as predominant symptom.

**TABLE 2:** Randomized controlled trials evaluating the efficacy peppermint oil in IBS patients with diarrhea.

Study	Population (*n*)*,* Rome criteria	Period (weeks)	Dose	Main outcome, significant difference compared to placebo	Adverse events
Liu et al. (1997), Taiwan	IBS (*n* = 101, 52 PO), No Rome criteria	4	187 mg t.i.d. or b.i.d	Alleviation of the severity in abdominal pain (RR: 79% vs. 43%)	Heartburn and mild transit skin rash
Cappello et al. (2007), Italy	IBS (*n* = 57, 28 PO), Rome II	4	450 mg b.i.d	Decrease >50% in total IBS symptom scores (RR: 75% vs. 38%)	Prolonged heartburn
Merat et al. (2010), Iran	IBS (*n* = 90, 33 PO), Rome II	8	187 mg t.i.d	Significant difference in the number of abdominal pain free patients (RR: 42% vs. 22%)	Heartburn, headache, and dizziness
Cash et al. (2016), United States	IBS-M and IBS-D (*n* = 72, 35 PO), Rome III	4	180 mg t.i.d	Reduction of the total IBS symptom scores (RR: 40% vs. 24%)	Dyspepsia, gastroesophageal reflux, flatulence
Weerts et al. (2020), The Netherlands	IBS (*n* = 189, 62 SBR-PO 63 ICR-PO Rome IV	8	182 mg t.i.d	No significant differences in abdominal pain	SBR-PO: heartburn, GERD, belching, headache; ICR-PO: Altered anal sensation or sensitive urethra, headache, abdominal cramps

b.i.d., twice daily; GERD, gastro-esophageal reflux disease; ICR-PO, ileo-colonic release-peppermint oil; IBS, irritable bowel syndrome; IBS-D, IBS with predominant diarrhea IBS-M, IBS with mixed bowel habits; o. d., once daily; PO: peppermint oil; RR, response rate; SBR-PO: small-bowel release-peppermint oil; t. i.d., thrice daily.

#### Loperamide Targeting Altered Bowel Habits

This μ-opioid-receptor agonist, decreasing the contractions of the smooth muscles in the intestinal wall, is specifically used to improve stool frequency and consistency in IBS-D patients (Ford et al., 2018b). By binding to its receptor and inhibiting acetylcholine and prostaglandins release, loperamide hydrochloride also affects the water and electrolyte movement through the intestinal wall (Ford et al., 2019). Furthermore, this first-line treatment for IBS-D also increases the anal sphincter tonus which improves symptoms such as, urgency and incontinence (Ford et al., 2018b). However, the evidence regarding this long-standing treatment option is limited and is more based on clinical practice than on high-quality RCTs. Outdated RCTs don’t provide significant differences in the improvement of overall IBS symptoms or abdominal pain when comparing placebo and loperamide for 3–13 weeks (Hovdenak, 1987; Lävo et al., 1987). Nevertheless, these findings did support an improvement in urgency, stool consistency, and frequency. Currently, due to the unfavorable outcomes, the long-term use of loperamide is often not authorized. Despite the rather low-quality evidence, the drug is frequently used to reduce acute diarrhea symptoms with a dose of approximately 2 mg o. d. or b. i.d. IBS-D patients use loperamide preventively to avoid the onset of diarrhea in certain situations that could exacerbate symptoms (Moayyedi et al., 2019). One should be aware of common side effects and contraindications. Side effects include abdominal pain, constipation, nausea, vomiting, and bloating. Torsade de pointes, prolonged QT intervals are the most important contraindications.

**Good candidates for loperamide (first-line)** Mild to severe IBS-D patients with diarrhea (*i.e.* high stool frequency, loose consistency, with or without urgency, fecal incontinence) as predominant symptom. Loperamide can also be used prophylactically in stressful situations.

### Second-Line Pharmacological Treatments

#### Antidepressants Targeting Persistent Abdominal Pain

Antidepressants are beneficial for IBS patients potentially due to their central effects, but most importantly due to their peripheral effects targeting underlying mechanisms, such as visceral hypersensitivity, pain perception, and motility (Ford et al., 2019). Antidepressants may alter the patient’s pain perception by modulating the visceral afferents via anticholinergic effects and by blocking incoming pain impulses (Ford et al., 2019). However, the precise mechanism of these agents in IBS and other abdominal pain related DGBIs is incompletely understood. Their beneficial influence on GI motility could be originating from their effects on the levels of neurotransmitters serotonin and norepinephrine and of the brain-gut peptides, including motilin, ghrelin, and neuropeptide-Y, which can regulate the secretory and motor functions of the GI tract (Huang et al., 2013). The classes of antidepressants that are most frequently used in IBS management are the tricyclic antidepressants (TCA), slowing down GI transit, and the selective serotonin reuptake inhibitors (SSRIs), accelerating transit (Ford et al., 2019). This explains why TCAs are mostly prescribed to IBS-D patients and SSRIs either to IBS-C patients or to IBS patients with predominant psychological comorbidities. In Table 3, you can find an overview of RCTs providing evidence for and against the use of TCAs and SSRIs in approximately 500 IBS patients in total.

**TABLE 3:** Randomized controlled trials evaluating the efficacy of TCA and SSRI in IBS patients with diarrhea.

Study	Population (*n*)*,* Rome criteria	Period, weeks	Dose	Main outcome, significant difference compared to placebo	Adverse events
**Imipramine (TCA)**					
Abdul-Baki et al. (2009), Lebanon	IBS (n = 107, 31 imipramine), Rome II	12	25–50 mg o.d	Significant difference in global symptom relief (RR: 81% vs. 48%)	Sleep disturbance, dizziness, urologic symptoms, anxiety, palpitations, dry mouth, flushing, constipation
**Amitriptyline (TCA)**					
Vahedi et al. (2008), Iran	IBS-D (n = 50, 25 amitriptyline), Rome II	8	10 mg o.d	Complete loss of all symptoms (RR: 68% vs. 28%)	Sleepiness, tachycardia, constipation, and blurred vision and dry mouth
**Imipramine (TCA) vs citalopram (SSRI)**					
Talley et al. (2008), Australia	IBS (n = 51, 17 citalopram, 18 imipramine), Rome II	12	25–50 mg o.d. vs. 20–40 mg o.d. (increase w3)	No significant difference in adequate IBS symptom relief	Abdominal pain, diarrhea, constipation, bloating, headache, and nausea
**Citalopram (SSRI)**					
Tack et al. (2006a), Belgium	Non-depressed IBS (n = 23, 11 citalopram), Rome II	6	20–40 mg o.d. (increase w4)	≥50% reduction of abdominal pain days (RR: 100% vs. 33%)	Nausea
Ladabaum et al. (2010), United States	IBS (n = 54, 27 citalopram), Rome II	8	20–40 mg o.d. (increase w5)	No significant differences in adequate symptom relief	Not reported
**Fluoxetine (SSRI)**					
Kuiken et al. (2003), Netherlands	IBS (n = 40, 19 fluoxetine), Rome I	6	20 mg o.d	No significant differences in rectal sensitivity or abdominal pain scores	Dizziness and drowsiness
**Paroxetine (SSRI)**					
Creed et al. (2003), United Kingdom	IBS (n = 171, 86 paroxetine), Rome I	64	20 mg o.d	Both improved the physical aspect of health-related quality of life	Sedation, light-headedness, sexual or sleep problems, nausea, and diarrhea

b.i.d., twice daily; IBS, irritable bowel syndrome; IBS-D, IBS with predominant diarrhea; o. d., once daily; RR, response rate; SSRI, selective serotonin re-uptake inhibitors; TCA, tricyclic antidepressants; t. i.d., thrice daily; w, week.

The dose of TCAs prescribed to improve IBS symptoms, is a dose below the prescribed concentration to treat psychiatric disorders (Clouse, 2003). Benefits of taking antidepressants can be seen after taking these agents for at least 1–3 months and effects can be long-lasting without tachyphylaxis. This difference in this dose, the treatment target, and delayed effect is something that needs to be clearly explained to the patient. The patient needs to be aware that antidepressants are prescribed to target severe and persistent chronic abdominal pain and not depression. Examples that are prescribed to IBS patients include imipramine, desipramine, amitriptyline, and its equivalent nortriptyline. However, when starting with these second-line medications, patients risk experiencing anticholinergic side effects, such as dry mouth and eyes, constipation, drowsiness, weight gain, and QT-interval prolongation (Ford et al., 2019). Usually, a few days or weeks after these side effects appear, they fade away. However, keeping a low and steady dose (especially during the first week to the first 3 months), is necessary to monitor these potential side effects. Contraindications include heart or liver disease, glaucoma, and epilepsy.

The dose of SSRIs prescribed to IBS patients represent the full psychiatric dose, used to reduce anxiety and depression (Tack et al., 2006a). The most often prescribed agents include citalopram, fluoxetine, paroxetine, escitalopram, sertraline, and venlafaxine. Findings support that the effect of SSRIs mostly originates from a decrease in psychiatric comorbidities that indirectly affect IBS symptoms (Creed, 2006; Creed et al., 2008). However, literature also indicates that there is an analgesic effect, supporting the fact that SSRIs do improve general IBS symptoms (mostly abdominal pain) independent of improved depression scores (Kuiken et al., 2005; Creed, 2006), in spite of one study assessing the efficacy of one specific SSRI (fluoxetine). They showed that fluoxetine did not improve rectal sensitivity (Kuiken et al., 2003). Furthermore, these second-line agents seem to decrease multiple bodily symptoms or somatization, and improve health-related quality of life. Side effects are less common for SSRIs compared to TCAs, but include dry mouth, nausea, drowsiness, insomnia, and hyperhidrosis. The use of SSRIs in patients with bleeding disorders, type 1 and 2 diabetes, kidney disease or epilepsy is contraindicated.

**Good candidates for antidepressants (second-line)** **TCAs** Moderate to severe IBS-D patients (with potential overlap of other pain-related DGBIs or with somatization) with persistent and/or severe abdominal pain as predominant symptom. **SSRIs** Moderate to severe IBS-D patients with psychological comorbidities. However, SSRIs are not frequently used in clinical practice, and most often considered when the usage of TCAs is contraindicated.

#### Cholestyramine Targeting Altered Bowel Habits

Twenty-five to 50% of the patients with IBS-D show signs of excess bile acids entering the colon or bile acid malabsorption (BAM) (Camilleri, 2015). BAM leads to the stimulation of secretion and motility and in turn to symptoms, such as loose or watery stools, urgency, and fecal incontinence, defined as bile acid diarrhea (Mekjian et al., 1971; Bampton et al., 2002; Mottacki et al., 2016). BAM leading to bile acid diarrhea often occurs after cholecystectomy. Usually, symptoms improve approximately 6 months after the intervention, but sometimes patients end up suffering from chronic diarrhea. Research show that 96% of the patients with chronic diarrhea suffer from BAM (Sciarretta et al., 1992). Ninety-two percent of BAM patients experience symptom improvement after being treated with bile acid binding agent, cholestyramine. Recent findings show that when the sequestrant is compared to hydroxypropyl cellulose, cholestyramine has a significantly greater effect in the number of watery stools (Fernández-Bañares et al., 2015). BAM can be recognized by decreased levels of fibroblast growth factor 19 in the serum (Camilleri, 2015). The diagnosis can also be made with the help of a fecal bile acid test, quantifying individual and total bile acids in 2-days stool collections or with the serum 7αC4 test, assessing serum C4 levels, which are elevated in patients with BAM (Mottacki et al., 2016). The test used most of the time in Europe is the 75selenium homotaurocholic acid 7-days retention test (SeHCAT), including a capsule with a synthetic analogue of the natural conjugated bile acid tauroselcholic acid and 75Se (a gamma-emitter). A gamma camera can be used to trace the radionuclide and therefore measure if the radionuclide is lost or retained in the feces. However, the SeHCAT test is only available at tertiary care centers in a limited number of European counties (and in Canada) and is relatively expensive. Often, clinicians test the efficacy of cholestyramine to diagnose, and sometimes simultaneously manage BAM without the result of a fecal bile acid or SeHCAT test.

**Good candidates for cholestyramine (second-line)** Mild to severe IBS-D patients with BAM or patients with diarrhea as predominant symptom (after loperamide failure or worsening of the symptoms after cholecystectomy).

#### Serotonin Receptor Antagonists Targeting Altered Bowel Habits and Abdominal Pain

**FIGURE 2:**
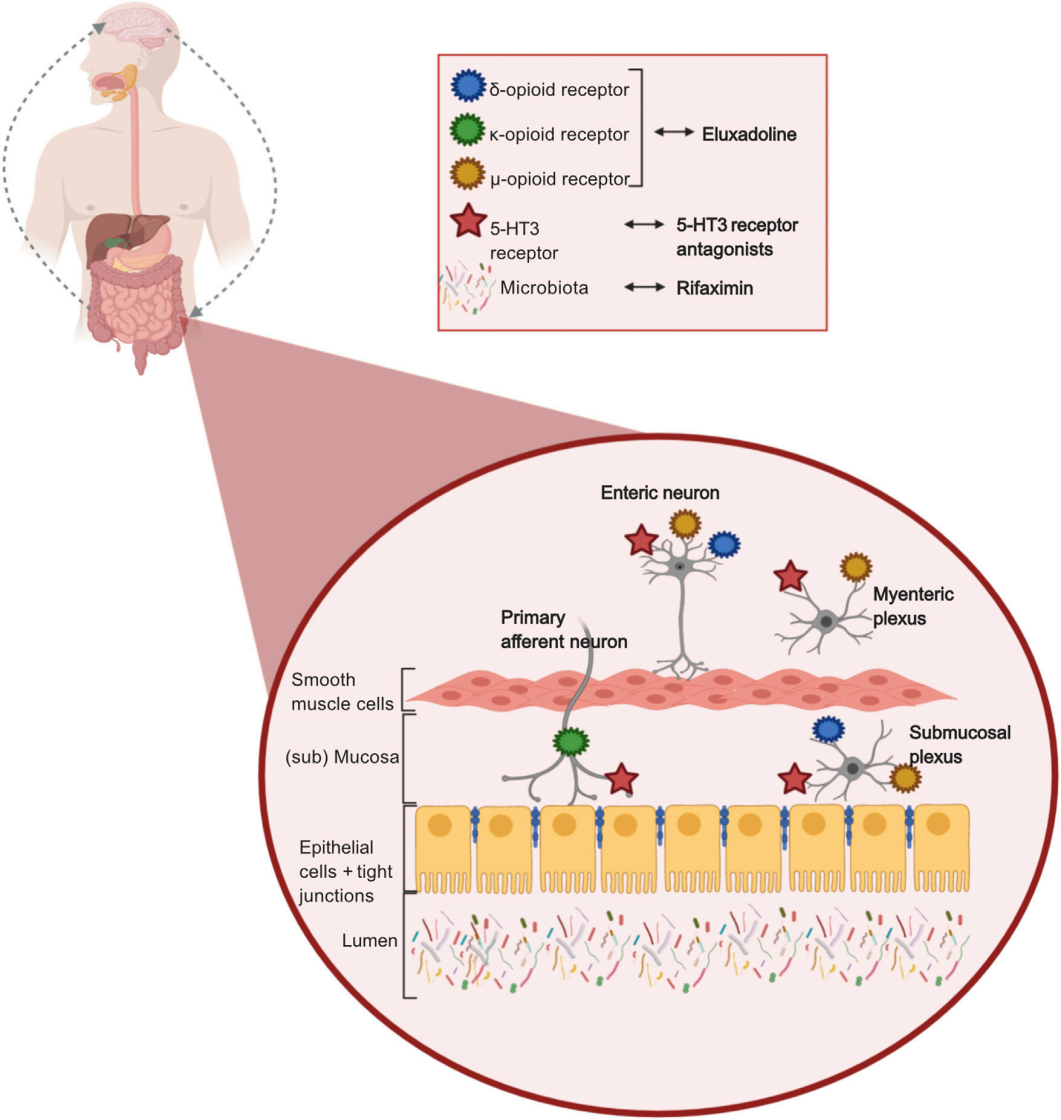
Mechanisms of action of pharmacological treatments in IBS-D. 5-HT_3_ receptor antagonists, targeting 5-HT_3_ receptors located on enteric neurons, myenteric plexus, submucosal plexus, and primary afferent neurons, reducing sensory signals, secrotory and motor reflex in the gut. Eluxadoline (opioid receptors agonist), targeting δ-,µ-, and κ-opioid receptors located on enteric neurons, myenteric plexus, submucosal plexus, and primary afferent neurons delaying transit by reducing secretory and sensory signals. Rifaximin, targeting the luminal gut microbiome due to its non-absorbable and non-systemic properties. Created with BioRender.com.

5-Hydroxytryptamine (5-HT), i.e., serotonin, is of importance in signaling pathways in the gut-brain interaction. Secretory and peristaltic reflexes in the gut are activated by 5-HT via primary afferent neurons. The 5-HT_3_ receptor is one of seven subtypes of the 5-HT receptors, and its main function is to stimulate release of neurotransmitters. Serotonin stimulates the 5-HT_3_ receptor to release acetylcholine in the nerve ends, which causes smooth muscle contraction and enhanced intestinal secretion (Marciani et al., 2010). Ondansetron, alosetron, and ramosetron are 5-HT_3_ receptor antagonists which inhibit the 5-HT_3_ receptor activation on the mucosal processes of the primary afferent neurons, and reduce activity of the secretory and motor reflex, by inhibiting the submucosal plexus and myenteric plexus respectively (Figure 2). 5-HT_3_ receptor antagonists also reduce depolarization of sensory neurons, which causes reduced sensory signals, which affects GI pain signals to the brain, and intestinal secretion. It has been demonstrated that 5-HT_3_ antagonists reduce abdominal pain, stool frequency, urgency, and increase stool consistency in patients with IBS-D (Zheng et al., 2017; Black et al., 2020a).

Multiple RCTs, in more than 3,700 IBS-D or IBS-M patients, have demonstrated that alosetron significantly reduces abdominal pain and improves stool consistency compared to placebo (Table 4). The severe complication ischemic colitis was reported incidentally, which first led to withdrawal of alosetron in the USA. However, trials in women have been done and suggested that alosetron is effective and safe to use (Olden et al., 2019). Therefore, alosetron is only available in a selected population: women with severe IBS in the USA, but still unavailable anywhere else in the world. In order to identify possible predictors of response, a pilot study investigated psychological distress and neural activity in IBS patients. They found that less activity in the orbitofrontal cortex (bilateral) and medial temporal gyrus predicted greater symptom improvement (Jarcho et al., 2008).

**TABLE 4:** Randomized controlled trials evaluating the efficacy of 5-HT_3_ receptor antagonists, rifaximin, and eluxadoline in IBS patients with diarrhea.

Study	Population (*n*)*,* Rome, women/men/mixed	Period (weeks)	Dose	Main outcome, significant difference compared to placebo	Adverse events
**Alosetron (5-HT_3_ receptor antagonist)**					
Krause et al. (2007), United States	IBS-D (*n* = 353, 177 alosetron), Rome II, women	12	0.5 o.d. or 0.5 or 1 mg b.i.d	Improved global symptoms in all groups (RR: 51% 0.5 mg o.d., 48% 1 mg o.d., 43% 1 mg b.i.d., and 31% placebo)	Constipation
Chang et al. (2005), United States	IBS-D (*n* = 386, 258 alosetron), Rome I, men	12	0.5 or 1 mg b.i.d	Adequate relief of global symptoms (RR: 53% vs. 40%)	Constipation ischemic colitis
Chey et al. (2004), United States	IBS-D (*n* = 569, 279 alosetron), Rome I, women	48	1 mg b.i.d	Adequate relief of global symptoms (RR: 52% vs. 44%)	Constipation
Camilleri et al. (2001), United States	IBS-D (*n* = 626, 309 alosetron), Rome I, men	12	1 mg b.i.d	Adequate relief of global symptoms (RR: 43% vs. 26%)	Constipation
Lembo et al. (2001), United States	IBS-D or IBS-M (*n* = 801, 532 alosetron), Rome II, women	12	1 mg b.i.d	Improved global symptoms (RR: 76% vs 44%)	Constipation
Camilleri et al. (2000), United States	IBS-D or IBS-M (*n* = 647, 324 alosetron), Rome I, women	12	1 mg b.i.d	Adequate relief of global symptoms (RR: 41% vs. 29%)	Constipation
Camilleri et al. (1999), United States	IBS-D or IBS-M (*n* = 152, 72 alosetron), Rome I, mixed	12	1, 2, 4, 8 mg b.i.d	Adequate relief of global symptoms in women (RR: 60% 1 mg, 59% 2 mg, 51% 4 mg, 52% 8 mg, and 33% placebo)	Constipation
**Ramosetron (5-HT**_**3**_ **receptor antagonist)**					
Fukudo et al. (2017), Japan	IBS-D (*n* = 305, 203 ramosetron), Rome III, women	12	1.25, 2.5 or 5 μg o.d	Improved abdominal discomfort and pain (RR: 64% vs. 41%)	Constipation
Fukudo et al. (2016), Japan	IBS-D (*n* = 576, 292 ramosetron), Rome III, women	12	2.5 μg o.d	Improved global symptoms (RR: 51% vs. 32%)	Constipation
Fukudo et al. (2014), Japan	IBS-D (*n* = 296, 147 ramosetron), Rome III, men	12	5 μg o.d	Improved stool consistency (RR: 50% vs. 20%)	Hard stools
Matsueda et al. (2008a), Japan	IBS-D (*n* = 212, 103 ramosetron), Rome II, mixed	12	5 or 10 μg o.d	Adequate relief of symptoms (5 μg 43%, 10 μg 43%, and placebo 27%)	Hard stools, constipation
Matsueda et al. (2008b), Japan	IBS-D (*n* = 539, 270 ramosetron), Rome II, mixed	12	5 μg o.d	Adequate relief of symptoms (RR: 47% vs. 27%)	Hard stools, constipation
**Ondansetron (5-HT**_**3**_ **receptor antagonist)**					
Plasse et al. (2020), United States	IBS-D (*n* = 126, 75 ondansetron), Rome III, mixed	8	12 mg o.d. (bimodal release)	Improved stool consistency (RR: 56% vs. 35%)	Constipation, flatulence
Garsed et al. (2014), United Kingdom	IBS-D (*n* = 120), Rome III, mixed	5 + 5	4 mg o.d	Cross-over study. Improved stool consistency, mean difference stool form (−0.9, 95% CI −1.1–-0.6)	Constipation
**Rifaximin (Antibiotics)**					
Pimentel et al. (2011), United States	IBS-D or IBS-M (*n* = 623, 309 rifaximin), Rome II, mixed	2	550 mg t.i.d	Improved global symptoms (RR: 41% vs. 32%)	No differences with placebo
Pimentel et al. (2011), United States	IBS-D or IBS-M (*n* = 637, 316 rifaximin), Rome II, mixed	2	550 mg t.i.d	Improved global symptoms (RR: 41% vs. 32%)	No differences with placebo
Lembo et al. (2016b), United States	IBS-D (*n* = 692^a^, 328 rifaximin), Rome III, mixed (repeat treatment)	2	550 mg t.i.d	More responders with improved global symptoms (RR: 38% vs. 32%)	Nausea
**Eluxadoline (opioid receptors agonist)**					
Brenner et al. (2019), Canada, United States	IBS- D (*n* = 346, 172 eluxadoline), Rome III, mixed	12	100 mg b.i.d	Improved global symptoms (RR: 23% vs 10%)	Nausea, pain, constipation, vomiting
Lembo et al. (2016a), United States, Europe	IBS-D (*n* = 1,282, 855 eluxadoline), Rome III, mixed	52	75 or 100 mg b.i.d	Improved stool consistency and abdominal pain, composite score (RR: 24% 75 mg, 25% 100 mg, and 17% placebo)	Nausea, pain, constipation, pancreatitis
Lembo et al. (2016a), United States, Europe	IBS-D (*n* = 1,146, 764 eluxadoline), Rome III, mixed	26	75 or 100 mg b.i.d	Improved stool consistency and abdominal pain, composite score (RR: 29% 75 mg, 30% 100 mg, and 16% placebo)	Nausea, pain, constipation, pancreatitis
Dove et al. (2013), United States	IBS-D (*n* = 348, 176 eluxadoline), Rome III, mixed	12	5, 25, 100, 200 mg b.i.d	Improved clinical response (RR: 12% 25 mg, 14% 200 mg, and 6% placebo)	Nausea, pain, constipation, pancreatitis

^a^ Responders to rifaximin 550 mg t. i.d. 2 weeks with relapse of symptoms within 18 weeks, were randomized in repeat treatment or placebo.

5-HT, 5-hydroxytryptamin; b. i.d., twice daily; CI, confidence interval; GI, gastrointestinal; IBS, irritable bowel syndrome; IBS-D, IBS with predominant diarrhea; IBS-M, IBS with mixed bowel habits; o. d, once daily; RR, response rate; t. i.d., thrice daily.

Ramosetron has also been studied extensively in close to 2,000 IBS-D patients (Table 4). So far, there are no indications that ramosetron causes serious adverse events such as ischemic colitis. Ramosetron especially improves abdominal pain in IBS-D and IBS-M, but is for now only available in Japan (and a few other Asian countries). In both alosetron and ramosetron, constipation is the most frequent reported adverse event, contraindications are severe constipation and other GI diseases e.g., inflammatory bowel diseases and colon carcinoma.

In the same class, ondansetron is an older treatment, commonly used in patients undergoing chemotherapy to reduce nausea and vomiting. It has not been used extensively in IBS-D, but recent research showed that ondansetron effectively reduced GI symptoms in this population (Garsed et al., 2014) (Table 4). A follow-up study, assessing rectal biopsies of the patients, found that patients with the lowest 5-HT concentration in the rectum, responded the greatest to ondansetron (Gunn et al., 2019). Moreover, a recent RCT indicated that bimodal release ondansetron, i.e., RHB-102, is a promising treatment in IBS-D (Table 4), with indications of C-reactive protein (CRP) as a predictor of response. Comparing subgroups, patients with higher levels of CRP (still in the normal range) seemed to have a better response to the treatment compared to patients with lower levels of CRP (Plasse et al., 2020), but this needs to be confirmed in larger trials. The studies did not report any contraindications. Thus, large clinical trials are needed to confirm and determine the effects of ondansetron and bimodal release ondansetron in IBS-D.

5-HT_3_ receptor antagonists seem to be effective in patients with IBS-D, a frequent adverse event is constipation, demonstrating that GI transit is effectively delayed. Unfortunately, alosetron and ramosetron are not widely available despite their potential side-effects. However, ondansetron which is available worldwide, seems to be a promising safe alternative for IBS-D patients, but large clinical trials are still needed.

**Good candidates for 5-HT_3_ receptor antagonists (second-line).** **Alosetron** Women with severe IBS-D (in the USA) with diarrhea or abdominal pain as predominant symptoms. **Ramosetron** Moderate to severe IBS-D patients (in Japan, and a few other Asian countries) with diarrhea or abdominal pain as predominant symptoms. **Ondansetron** Moderate to severe IBS-D patient with diarrhea and/or bloating as predominant symptoms.

## Rifaximin Targeting Bloating

Rifaximin, which is a broad-spectrum, non-systemic oral antibiotic, specifically targets the gut microbiome. Thus, it is likely that the possible mechanism of action is gut microbiota modulation (Figure 2). However, data from a study in rats indicates that rifaximin prevents putative pathophysiological mechanisms (i.e., impaired gut permeability, visceral hyperalgesia, and low-grade inflammation) induced by stress as well (Xu et al., 2014).

Three large RCTs (in total almost 2,000 IBS-D or IBS-M patients) have investigated the effects of rifaximin in patients with IBS-D (Table 4). They concluded that rifaximin 550  mg t. i.d. improved global IBS symptoms, especially bloating in IBS patients compared to placebo. However, the differences in response between rifaximin and placebo were modest. No effects were seen on stool consistency, and adverse events were not different from placebo (i.e., constipation), except for nausea (Lembo et al., 2016b). One trial found that rifaximin was effective and safe to repeat in IBS-D patients that relapsed after an initial effective treatment (Lembo et al., 2016b). However, a recent systematic review and meta-analysis concluded that rifaximin failed to achieve a response in global IBS symptoms and abdominal pain (Black et al., 2020a). On the contrary, all trials suggest that rifaximin effectively relieves bloating in IBS-D patients (Pimentel et al., 2011; Lembo et al., 2016b). Additionally, one study investigated the possibility of lactulose breath testing as a predictor of response to rifaximin. They found that IBS-D patients with a positive baseline lactulose breath test, had a higher likelihood of responding to rifaximin (Rezaie et al., 2019), but this needs to be further investigated in larger studies.

Unlike 5-HT_3_ receptor antagonists, constipation was not reported as an adverse event in the trials assessing rifaximin. It has been demonstrated that rifaximin actually increases colonic transit time in non-constipated IBS patients (Acosta et al., 2016). Therefore, the only contraindication is a history of obstruction in the GI tract. The exact mechanism why a subgroup of IBS patients respond to rifaximin is not known. Due to its safety and absence of adverse events, rifaximin is a suitable first- or second-line treatment option for patients with IBS-D with predominant bloating, comorbidities or contraindications to other treatments.

**Good candidates for rifaximin (first- or second-line)** Moderate to severe IBS-D patients (with or without SIBO) with bloating as predominant symptom.

## Eluxadoline Targeting Stool Consistency and Abdominal Pain

Like loperamide, eluxadoline activates μ-opioid receptors in the gut, causing delayed transit and treating diarrhea. Eluxadoline activates not only the μ-opioid receptors, but also the κ-opioid receptors and the δ-opioid receptors (Figure 2), involving secretion and sensation (Bitar and Makhlouf, 1982).

So far, eluxadoline has been investigated by four large RCTs, assessing more than 3,100 IBS-D patients (Table 4), with promising results. In three trials, eluxadoline improved stool consistency at 12 weeks, but the differences compared to placebo were modest (Dove et al., 2013; Lembo et al., 2016a). A post-hoc analysis was done assessing two phase III studies, from Lembo *et al.*, they found that the response in the early phase of the trial, could predict the therapeutic benefit after a follow-up of six months (Chey et al., 2017). One trial also investigated patients that did not respond to initial loperamide treatment, and found also that IBS-D patients refractory to loperamide had improved stool consistency and abdominal pain with eluxadoline (Brenner et al., 2019). Frequent adverse events were constipation, nausea, and vomiting. The dose of 100 mg b. i.d. was effective for all endpoints in all the studies. However, there are concerns regarding safety. Pancreatitis and sphincter of Oddi spasms were observed in multiple individuals, especially in patients that underwent cholecystectomy prior to the study. Therefore, eluxadoline is contraindicated in patients with biliary duct obstruction, history of cholecystectomy, alcoholism, pancreatitis, and hepatic impairment. Thus, eluxadoline is a suitable option for non-constipated IBS patients with prior failure of loperamide.

**Good candidates for eluxadoline (second-line)** Women and men with moderate IBS-D, and both abdominal pain and diarrhea as predominant symptoms, after loperamide failure and without any contraindication (cholecystectomy, biliary duct obstruction, pancreatitis, hepatic impairment, alcohol abuse, and chronic or severe constipation).

## Probiotics and Plant-Derived Products

Probiotics are not considered as pharmacological treatments, but due to their good accessibility, their popularity among patients, and applicability in IBS-D patients with contraindications, we included probiotics in this review. Recently, plant-derived products have also emerged as treatment options in IBS-D. Note that these are also not pharmacological treatments, but they are worth mentioning as options for patients with comorbidities or contraindications for other pharmacological treatments.

### Probiotics Targeting the Dysbiosis Between the Host and the Microbiota

Probiotics are living bacteria that confer a health benefit to the host when consumed in sufficient quantities. For decades, IBS patients have been using probiotics on an empirical basis, due to their suggested beneficial effect on dysbiosis. Researchers observed differences in the microbial compositions of IBS patients compared to healthy controls (Tap et al., 2017). When alterations in the homeostatic state appear, the tolerance among commensal microbes that maintain symbiotic functions as well as the barrier integrity cannot be persevered. Due to a potential alteration in the immune response, pathogens can easily provoke inflammation which will in turn affect the gut luminal environment and its microbial composition. Thus, dysbiosis which is a microbial imbalance with a reduced microbial diversity can arise (Pédron and Sansonetti, 2008; Chong et al., 2019). However, a comprised epithelial barrier and an altered immune activation are not the only factors implicated in the IBS pathophysiology and the microbial alterations seen in IBS patients. Post-infectious alterations, dietary changes, altered stress levels, low-grade mucosal inflammation with visceral hypersensitivity, and motility disturbances are all factors influencing each other and the onset of IBS symptoms that may or may not be linked to dysbiosis individually (Chong et al., 2019; Pimentel and Lembo, 2020).

**FIGURE 3:**
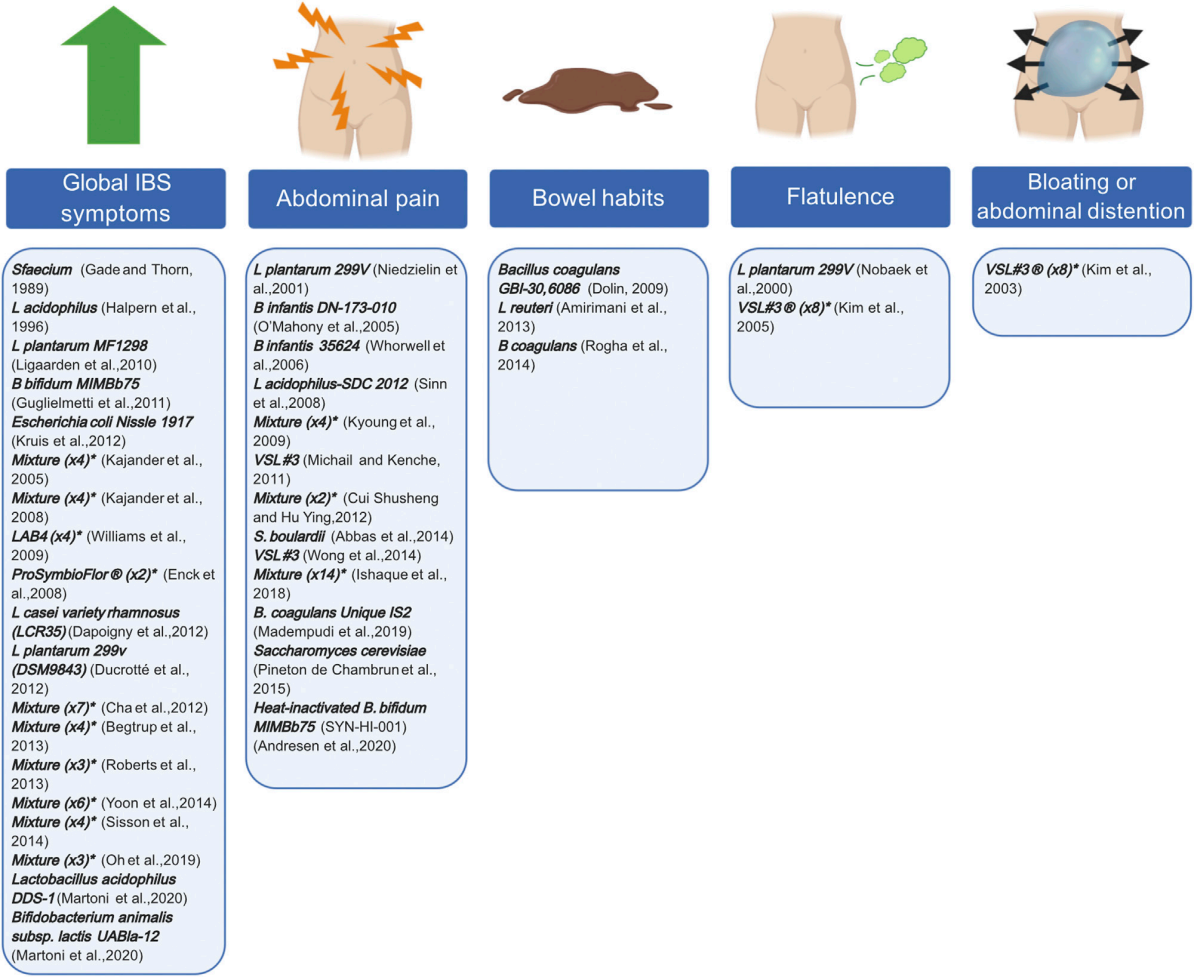
Probiotics and their symptom target in patients with IBS. Different probiotics, assessed in former and more recent RCTs, can target individual IBS symptoms. RCTs either demonstrated a significant effect on global IBS symptoms, abdominal pain, bowel habits, flatulence, bloating or abdominal distention or a combination thereof. * indicated the number of bacterial strains included in the mixture. Created with BioRender.com.

Probiotics are a rather old treatment option for IBS, but recently more and more research groups are interested in their specific targets (Didari et al., 2015; Zhang et al., 2016). Proposed targets include the dysbiotic microbial composition, small intestinal bacterial overgrowth (SIBO) that is often associated with IBS, and post-infectious alterations (Simreń et al., 2013; Chong et al., 2019). Interpreting the results of these probiotic studies, remains a continuous challenge due to the variety of the available species, strains, doses, duration, repetition, preparations and targeted patient populations. Furthermore, due to their short lifespan, patients need repeated doses to experienced adequate benefit. Meta-analyses suggested a superior role for *Lactobacillus* and Bifidobacterium compared to placebo, when it comes to reducing global IBS symptom scores and abdominal pain (Simreń et al., 2013). However, a trend seen in these meta-analyses is that higher-quality studies tend to demonstrate less of a treatment effect. Consequently, recommendations regarding individual species, preparations, or strains are difficult to make at this moment. Figure 3 describes examples of different probiotics that have been tested in RCTs and which GI symptom(s) they are targeting.

### Plant-Derived Products Targeting Stool Consistency and Abdominal Pain

A recent double-blind, placebo-controlled cross-over trial (*n* = 30, duration 4 + 4 weeks) investigated the efficacy of a recent plant-based medical device, which is intended to protect the intestinal mucosa, and thereby relieve GI symptoms and prevent diarrhea (Trifan et al., 2019). The components are a combination of pea protein, tannins (derived from a grape seed extract), xylo-oligosaccharides, and xyloglucan (*i.e.* tamarind seeds). The proposed mechanism of xyloglucan is that it forms a physical barrier, due to its mucin-like structure, that can protect the gut mucosa against proinflammatory components (e.g. food components), microorganisms or allergens (Piqué et al., 2018). IBS-D patients reported more BSFS type 3 and 4 stool types compared to placebo (response rate 90 vs. 12%), and that abdominal pain and bloating were more acceptable. No adverse events or contraindications were reported (Trifan et al., 2019).

Another promising plant-derived product is Crofelemer, which is extracted from stem bark latex of the *Croton* lechleri tree (Cottreau et al., 2012). Crofelemer was initially used in primary secretory diarrheal disorders, e.g., travelers’ diarrhea, cholera, and acute GI infections. A large RCT in IBS-D investigated the efficacy of Crofelemer in 240 patients, and found no differences in the primary outcome of stool consistency. However, they did find that women had improvement of pain- and discomfort-free days compared to placebo, no differences were seen in men (Mangel and Chaturvedi, 2009). Another large RCT (also in 240 IBS-D patients) aimed to investigate the analgesic properties of Crofelemer in women. They found that there was no significant difference in abdominal pain improvement (primary endpoint) between Crofelemer and placebo. However, they did find that Crofelemer significantly improved abdominal pain on the FDA monthly responder endpoint (Nee et al., 2019). Hence, large clinical trials are needed to investigate these plant-derived products further. Currently, we can not strongly recommend these options due to the absence of high-quality evidence.

**Good candidates for probiotics or plant-derived products (first- and second-line)** **Probiotics** Mild IBS patients with abdominal pain or bloating/abdominal distension as predominant symptom. Due to their safety profile, probiotics are a good option for pregnant or lactating women or patients with long term usage of antibiotics. **Xyloglucan + Pea protein = Tannins** Women and men with IBS-D with mainly liquid stools (BSFS 6–7) with bloating and/or flatulence. Patients with comorbidities could safely use this treatment. **Crofelemer** Women with IBS-D with abdominal pain as predominant symptom. Patients with comorbidities could safely use this treatment.

## Conclusion

As described previously, multiple incompletely elucidated pathophysiological mechanisms are involved in IBS. This has resulted in a wide range of pharmacological treatments, with heterogeneous treatment responses in IBS-D patients. Besides existing treatments, many recent treatments are still being discovered. We have reviewed the first- and second-line pharmacology targeting predominant IBS symptoms, as well as probiotics and plant-derived products. Choosing the right treatment for the right patients, remains a challenge for clinicians. A fundamental cause for this challenge is the heterogeneity in the IBS-D population and therefore, the inability of finding the specific mechanism that is causing the symptoms that need to be targeted in individual patients. Furthermore, the differences between pharmacological treatments and placebo (Tables 1–4) are often modest. This is most probably again caused by the heterogeneity between the patients in the disorder. Moreover, a placebo response is common in clinical trials in patients with IBS (Ford and Moayyedi, 2010).

Another challenge for clinicians is the low efficacy level of the available pharmacological treatments shown in clinical practice, and outdated RCTs in patients with IBS-D (Ford et al., 2008; Ford et al., 2014). First-line therapy is often chosen by healthcare professionals because of its wide availability instead of its specific target. Moreover, the availability of the more recent drugs is scarce in different geographical areas. At the moment, authorization of pharmacological treatments is different between countries. For example, in some countries in Europe high-quality evidence is required, where the more recent treatment is compared to an older pharmacological alternative, but this not the case in all countries (Authorisation of medicines, 2020). The data of the systematic review and meta-analysis show that both alosetron and ramosetron are most efficacious in IBS-D compared to placebo, but they are solely available with a restricted prescription (women with severe IBS-D) in the USA and Japan (and a few other Asian countries) respectively (Black et al., 2020a). For alosetron, incidental cases of ischemic colitis, with a low-prevalence risk (Ford et al., 2009), has caused unavailability for men with IBS-D. An explanation for the unavailability is that treatment-related adverse events are not well accepted in IBS, due to less morbidity and no increased risk of mortality compared to other disorders or diseases (Staller et al., 2020). As previously reviewed, the more effective treatments seem to have more adverse events. Currently, there are more possibilities available in safe supplements (*e.g.* probiotics and plant-derived products), but high-quality evidence is scarce (Ford et al., 2018a). These supplements are (usually) inexpensive and assessed for safety but, they are not assessed as pharmacological treatments by government institutes (e.g., Food and Drug Administration, USA), where also strict efficacy is needed to get approval. Thus, healthcare professionals should be reserved when it comes to recommending these supplements even though they are often highly preferred by patients. Currently, high-quality evidence regarding which supplement is targeting which IBS symptom is lacking. Therefore, further research is needed.

**FIGURE 4:**
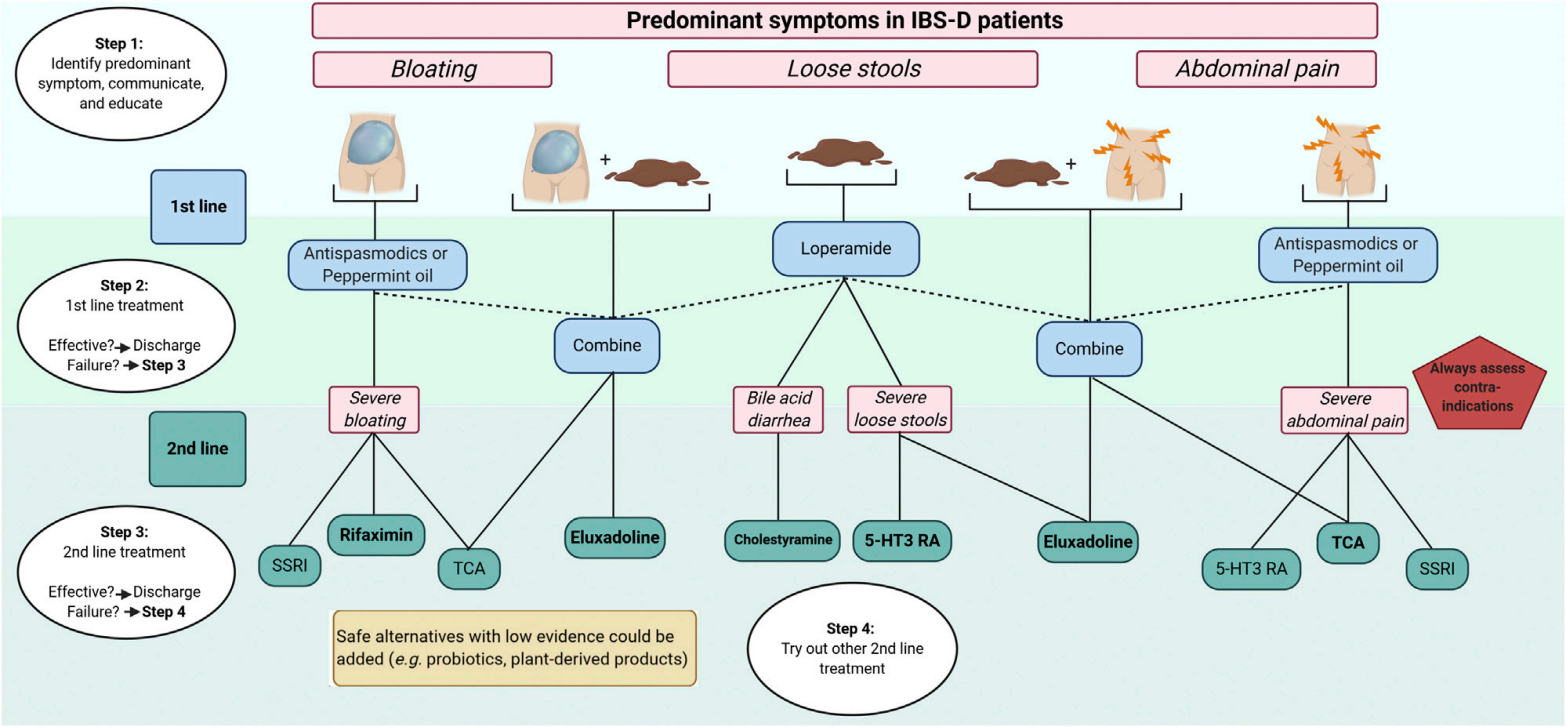
Predominant symptom-based algorithm for pharmacological treatments in IBS-D 5-HT3-RA, 5-hydroxytryptamine-3 receptor antagonists; IBS, irritable bowel syndrome; IBS-D, IBS with predominant diarrhea; TCA, tricyclic antidepressants; SSRI, serotonin re-uptake inhibitors. Created with BioRender.com.

A more personalized approach in the management of patients with IBS is desirable, and for now, targeting the most bothersome predominant symptoms in IBS-D (i.e., loose stools, abdominal pain, and/or bloating) seems to be the only practical and suitable treatment strategy. Not only preferences and history of the patient needs to be taken into consideration, but also healthcare associated costs, which are substantial (Nellesen et al., 2013). Successful pharmacological management usually starts with a good relationship between the healthcare professional and the patient. Treatment options should be discussed, as well as side effects. Key factors in the management of IBS-D patients are proper education about the disorder and treatments (Ringström et al., 2012; Lindfors et al., 2020), as well as explaining the reasons to do (or not do) investigations (e.g., colonoscopy) (Black and Ford, 2020). Moreover, besides pharmacology, initial simple dietary- and life-style advice is required (Algera et al., 2019), and effective psychological treatments (e.g., hypnosis and cognitive behavioral therapy) are available as well (Ford et al., 2014). Due to the scope of this review and the current challenges in identifying individual pathophysiological factors in IBS patients, we propose a predominant symptom-based approach solely focused on pharmacological management (algorithm, Figure 4). First-line pharmacology includes loperamide, peppermint-oil, and antispasmodics. Even if the level of proof is low, they are safe and could be used for a long time. With failure to improve symptoms, second-line treatment should be initiated. It remains important that clinicians check treatment failures. Obvious reasons (i.e., inadequate treatment period or practical usage) should be excluded. Second-line treatment includes 5-HT_3_ receptor antagonists, central neuromodulators (including TCA and SSRI) as well as recent opioid receptors agonists (eluxadoline). Bile-acid sequestrants can be chosen for patients with suspected (or confirmed by ^75^SeHCAT retention) BAM. Due to the unavailability of recent 5-HT_3_ receptor antagonists in some countries, ondansetron might be a suitable and safe alternative. Supplements, including probiotics and plant-derived products, are safe to use in clinical practice but, high-quality evidence of efficacy is currently lacking.

## Future Perspectives

Pharmacological treatments targeting the gut-brain interaction seem to be effective in patients with IBS-D (Ford et al., 2019), but available literature is not always of high quality. Moreover, there is a lack of clinical trials comparing pharmacological treatments. Therefore, large clinical trials are needed to assess and compare the efficacy of these treatments, ideally in a double-blinded, randomized, parallel design. Furthermore, future studies should focus on identifying predictors for treatment responsiveness, including comorbidities (e.g., anxiety and depression) and possible biological markers. As previously discussed, some studies demonstrate promising results, assessing predictors of response (Jarcho et al., 2008; Plasse et al., 2020). Future studies should reinvestigate these predictors of response in larger studies, and also focus on potential others. This will possibly enable the individual tailoring of pharmacological treatment options in patients with IBS-D.
